# Construction and application of multiple nucleotide polymorphism-based DNA fingerprinting for *Polygonatum cyrtonema* identification

**DOI:** 10.3389/fpls.2026.1758042

**Published:** 2026-02-27

**Authors:** Min Tang, Yuqing Long, Juan Zeng, Wei Xiang, Peng Huang, Gang Wang, Jianguo Zeng

**Affiliations:** 1 Department of Pharmacy, Yiyang Medical College, Yiyang, China; 2 Chinese Medicinal Materials Breeding Innovation Center, Yuelushan Laboratory, Changsha, China; 3 College of Horticulture, Hunan Agricultural University, Changsha, China; 4 Wuhan Mingliao Biotechnology Co., Ltd., Wuhan, China; 5 Hunan Key Laboratory of Traditional Chinese Veterinary Medicine, Hunan Agricultural University, Changsha, China; 6 College of Veterinary Medicine, Hunan Agricultural University, Changsha, China

**Keywords:** construction and application of technology, DNA fingerprinting, genetic marker, multiple nucleotide polymorphism (MNP), polygonatum cyrtonema, species identification

## Abstract

**Introduction:**

*Polygonatum cyrtonema* Hua is an important economic crop with dual use as food and medicine. Its market demand has been increasing steadily. However, in the current market, widespread cultivar confusion coupled with the lack of efficient and accurate cultivar identification methods has severely hindered the genetic improvement and standardized development of its industry. In this study, we aimed to address the issues of germplasm resource confusion and difficulty in cultivar discrimination of *P. cyrtonema* while providing a novel technical tool for investigating the genetic diversity of *Polygonatum* plants.

**Methods:**

We successfully developed a high-throughput identification system based on multiple nucleotide polymorphism (MNP) markers for *P. cyrtonema* cultivar identification. Via genome sequencing of 30 representative accessions, we screened 505 core MNP loci with high polymorphism and further optimized and established stable protocols for multiplex polymerase chain reaction amplification and high-throughput sequencing.

**Results:**

Validation results revealed that this marker panel had excellent amplification efficiency and polymorphism across all 80 tested accessions. For the MNP markers, the average number of allelic genotypes reached 28.95 ± 15.11. The polymorphism information content was 0.73 ± 0.16. Both metrics were substantially superior to those of traditional identification methods. Both phylogenetic analysis and principal component analysis distinguished all the tested accessions, with an identification accuracy rate of 99.90%. A unique molecular ID code was assigned to each cultivar.

**Discussion:**

The MNP marker system developed in this study combines the advantages of high throughput, high accuracy, and favorable reproducibility. It provides the technical means and solutions for the authenticity identification of *P. cyrtonema* cultivars, purity detection of seeds and seedlings, conservation of germplasm resources, as well as the conduct of distinctness, uniformity, and stability testing for new plant varieties and the application for new variety protection rights. These findings are expected to be applied for the source authentication of medicinal plants and population genetics research.

## 1 Introduction

Polygonati Rhizoma, known as Huangjing by its common commercial name and recorded in ancient Chinese medical classics, is a rhizomatous material with dual use as medicine and food. It has long been used as a tonic and a medicinal agent for treating various conditions, including weakness, dyspepsia, diabetes mellitus, and premature graying of hair in China, India, South Korea, Japan, and other Asian countries (Zhao et al., 2018, 2023). The main bioactive components of Huangjing include polysaccharides, saponins, that is, steroidal saponins and triterpenoid saponins, flavonoids, phenols, and lectins. These components have a range of pharmacological properties, such as anti-aging, anti-diabetic, immunomodulatory, antioxidant, anti-fatigue, anti-cancer, and anti-osteoporotic activities (Cui et al., 2018; Liu et al., 2022; Luo et al., 2022; Gong et al., 2023; Zhang et al., 2024). Polysaccharides play a particularly prominent role in regulating the immune system and treating diabetes (Zhao et al., 2019b; Liang et al., 2021). Due to its unique fructans and abundant nutrients, such as proteins, amino acids, vitamins, and minerals, Huangjing has been widely applied in multiple fields, including medicine, food, such as staples in healthy diets, candied products, tea, beverages, and fruit wines, cosmetics, and horticulture (Wujisguleng et al., 2012; Cheng et al., 2021; Shi et al., 2023; Chen et al., 2024), Therefore, it has considerable development potential and economic value. In China, the National Innovation Alliance for the Huangjing Industry was established, comprising 99 relevant entities such as research institutions, universities, and enterprises. The alliance aims to promote technological innovation and sustainable development of the Huangjing industry. By 2024, the output value of Huangjing in China had exceeded 20 billion yuan (RMB), and the industry is expected to grow into a 100-billion-yuan (RMB) market in the future.

*Polygonatum cyrtonema* Hua (*P. cyrtonema*) is one of the three botanical sources of Huangjing officially recorded in the Chinese Pharmacopoeia (2025 Edition). Among these sources, *P. cyrtonema* stands out for having the largest cultivation area, best quality, and highest market recognition. It is widely cultivated under forest canopies across more than 10 provinces and municipalities in China, including Hunan, Chongqing Municipality directly under the Central Government, Guizhou, Hubei, and Sichuan (Wang et al., 2023). With the surge in market demand for Huangjing, the scale of artificial cultivation of *P. cyrtonema* has been continuously expanding. However, this growth has also brought forth two critical challenges to the Huangjing industry. First, extensive over-harvesting of wild *P. cyrtonema* resources, combined with confusion in cultivated varieties and a lack of high-quality germplasm resources, has posed severe challenges to the conservation and sustainable use of the *P. cyrtonema* germplasm (Wang et al., 2022; Xia et al., 2022). Second, *P. cyrtonema* exhibits frequent interspecific hybridization and has high genetic variation. Including within the same region, plants growing in different locations may have substantial morphological variations (Xie et al., 2023), for instance, oblong or broadly ovate leaves (Ji et al., 2020), as well as green stems and purple stems. During the cultivation process, farmers often mistake *P. cyrtonema* for non-medicinal species of the *Polygonatum* genus. This severely impacts the safety and effectiveness of clinical medication (Ali et al., 2021). Therefore, researchers have conducted extensive studies on variety differentiation within the *Polygonatum* genus and the identification of *P. cyrtonema*. Classification of the *Polygonatum* genus or *P. cyrtonema* has been performed based on the morphology of plant leaves, rhizomes, or pollen grains (Ali et al., 2021; Ding et al., 2024). A variety of molecular marker technologies have also been developed and applied in fields such as taxonomic identification, phylogeny, and genetic diversity analysis of *Polygonatum* plants, including methods such as InDel (Ren et al., 2025), SRAP (Feng et al., 2020), SCoT (Jiao et al., 2018; Zhu et al., 2025), ISSR (Jiao et al., 2018; Liu et al., 2020), and simple sequence repeat (SSR) (Ji et al., 2020; Liu et al., 2023a; Pan et al., 2024; Hai et al., 2025), DNA barcoding (Zhou et al., 2020; Xie et al., 2023; Yao et al., 2025), and the combined analysis of morphology and molecular marker technology (Yang et al., 2024). The traditional evaluation methods for *Polygonatum cyrtonema* have inherent drawbacks, such as low accuracy and poor reproducibility. Although current molecular markers offer advantages, including high resolution and environmental stability, their identification scope is limited to the accessions collected in specific experiments. When a larger number of samples are analyzed, these markers typically exhibit low polymorphism and incur substantial economic costs. The markers developed to date have also failed to meet the requirements for precise germplasm resource management and medicinal quality control of *P. cyrtonema*. Therefore, the further development of low-cost, convenient, efficient, and stable molecular marker technologies represents a key breakthrough for realizing rapid cultivar identification of *P. cyrtonema*.

As an emerging molecular marker technology, MNP markers can detect variations in multiple adjacent nucleotides simultaneously. This technology combines the advantages of high polymorphism, high throughput, and low cost, and has already shown considerable advantages and potential in cultivar identification of some economic crops. Compared with existing molecular markers, MNP marker loci, obtained via whole-genome high-throughput sequencing and multiplex polymerase chain reaction (PCR) screening of dozens or even hundreds of samples, have shown exponential growth in diversity. This, in turn, provides greater discriminatory power for capturing diverse alleles in complex plant populations. In recent years, MNP marker methods have been developed and successfully applied to the identification of plant varieties, including those of grape (Liu et al., 2024a), *Chrysanthemum* (Liu et al., 2024b), and edible fungi, including *Lentinula edodes* (Ling et al., 2023), Enoki mushroom (Liu et al., 2023b), *Stropharia rugosoannulata* (Liu et al., 2025b), and *Agaricus bisporus* (Liu et al., 2025a). They have also been used in meat product origin identification (Yi et al., 2025) and detection of microbial genetic variations (Li et al., 2025). However, the use of MNP markers has, to the best of our knowledge, not yet been reported in medicinal plant classification and identification.

In this study, we focused on *P. cyrtonema* and developed its species-specific MNP marker loci based on high-throughput sequencing technology. Combined with molecular biology and bioinformatics approaches, we further evaluated the accuracy and applicability of these MNP markers in cultivar identification. By screening polymorphic loci and constructing a molecular identification system, we aimed to address the issues of germplasm resource confusion and difficulty in cultivar discrimination of *P. cyrtonema*, while providing a novel technical tool for investigating the genetic diversity of *Polygonatum* plants. This research contributes to promoting the scientific conservation and strategic use of *P. cyrtonema* germplasm resources and offers theoretical support for the quality standardization of Chinese medicinal materials, molecular breeding of medicinal plants, and the application and protection of patents for medicinal cultivars. Therefore, it has considerable implications for the sustainable development of the traditional Chinese medicine industry.

## 2 Materials and methods

### 2.1 Plant materials

**Figure 1:**
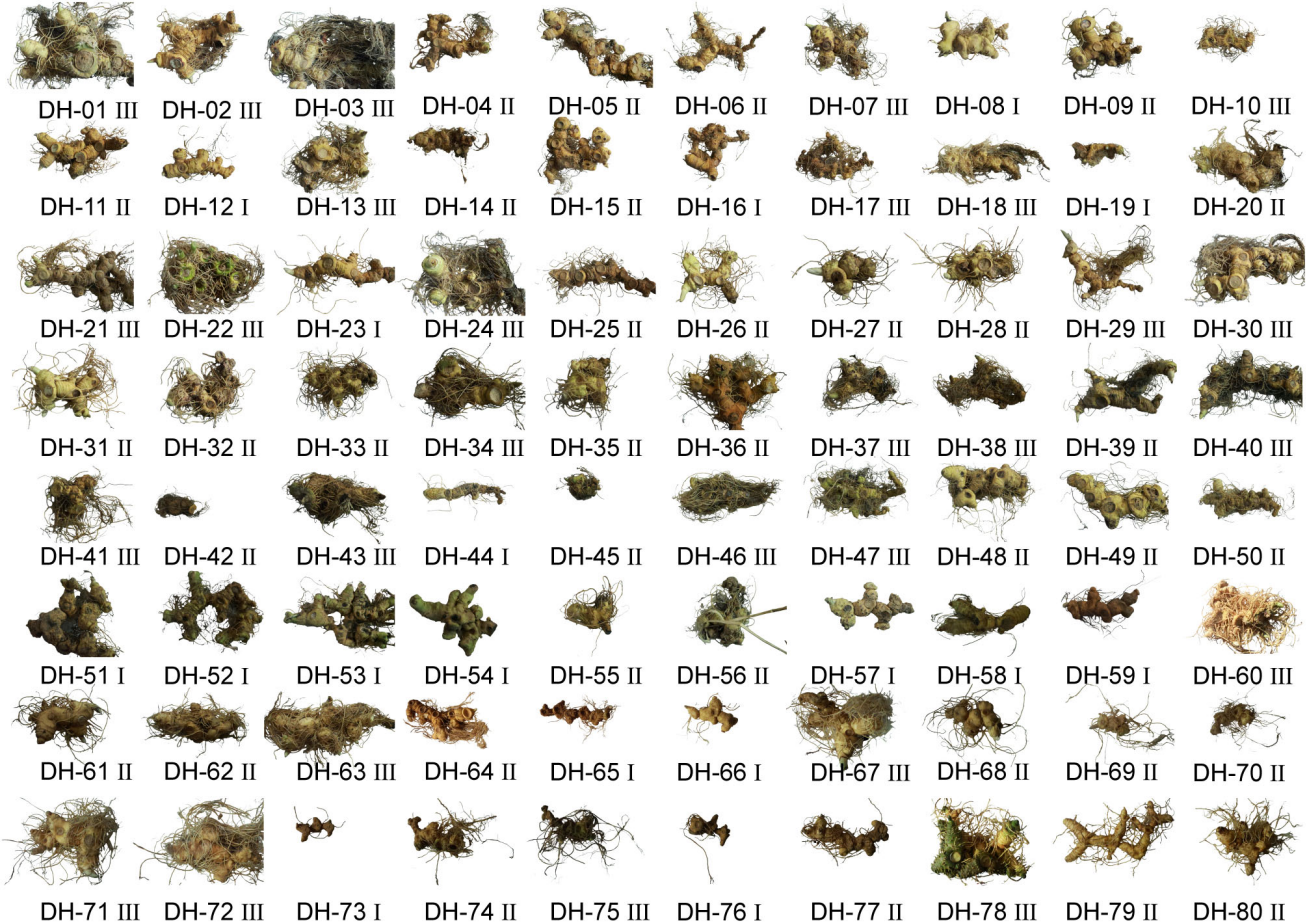
Eighty accessions of *Polygonatum cyrtonema* Hua were used in this study, designated as DH-01 to DH-80. Roman numerals I, II, and III denote sparse, moderately dense, and dense root systems, respectively.

Eighty *P. cyrtonema* accessions, encompassing both wild and cultivated types with broad geographical origins and diverse phenotypic traits, were collected in December 2023 from multiple provinces in China, including Hunan, Hubei, Sichuan, Guizhou, Anhui, Yunnan, Jiangxi, and Guangxi (**Figure 1**). These samples can be roughly divided into three categories based on root system density; however, they are difficult to distinguish by phenotypic traits (**Supplementary Figure S1**). These accessions were individually coded from DH-01 to DH-80 for traceability. All the samples were taxonomically identified as *P. cyrtonema* by Professor Zhou Ribao and Associate Professor Wang Zhi from Hunan University of Chinese Medicine, and Associate Professor Tang Min from Yiyang Medical College.

The collected accessions were planted under the forest canopy of the Germplasm Resource Nursery at Yiyang Medical College, with geographic coordinates of 42°20′34″ N, 116°31′49″ E. Fresh young leaves were collected separately from each accession for genomic DNA extraction. Thirty representative accessions were selected from the 80 materials for reduced-representation genome sequencing (RRGS). The 30 selected lines were derived from the major geographic production areas across eight provinces in China and exhibit abundant phenotypic diversity (variations in root system density, leaf shape and flowering time). Detailed information on these selected accessions is provided in **Supplementary Table S1**. As *P. cyrtonema* lacks a reference genome, whole-genome sequencing (WGS) was performed on accession DH-57 to support the screening of MNP markers.

### 2.2 DNA extraction and genome sequencing

Genomic DNA was extracted from the leaves of *P. cyrtonema* samples following the operating instructions of the Plant Genomic DNA Extraction Kit (Beijing Tiangen Biotech Co., Ltd., Beijing, China). For each sample, 1 μL of DNA was subjected to concentration and quality determination using a Qubit Fluorometer (Thermo Fisher Scientific Inc., Waltham, MA, USA). All the DNA samples were standardized to achieve a uniform initial concentration, after which they were digested with restriction enzymes Mse I and EcoR I-HF. The digested products were purified using GenoPrep DNA Clean Beads (Vazyme Biotech Co., Ltd., Nanjing, China).

Sequencing adapters (P5 and P7) were ligated to the purified digested products using T4 DNA ligase. The mixture was incubated at 22 °C for 60 min to construct the pooled sequencing library. The library was then recovered using a gel extraction kit, followed by further purification using a bead-based method. A 1 μL aliquot of the purified library was analyzed using the Qubit Fluorometer to assess its quality. Qualified libraries were subjected to high-throughput sequencing (HTS) on the Illumina NovaSeq 6000 platform at Novogene Bioinformatics Technology Co., Ltd. (Beijing, China).

Genome assessment was performed using K-mer analysis software (https://github.com/refresh-bio/KMC). Soapnuke software(https://github.com/BGI-flexlab/SOAPnuke) was used to filter out reads containing adapter sequences, low-quality reads, and reads with a proportion of N bases exceeding 1%. *De novo* assembly of *P. cyrtonema* reference contig sequences was conducted using SPAdes (version 3.13.0). Only contig sequences with a length > 250 bp were retained for the subsequent analyses.

### 2.3 Screening of MNP marker loci in *P. cyrtonema*

Genomic data from 30 representative *P. cyrtonema* accessions were aligned to the reference genome. Based on the alignment results, single-nucleotide polymorphisms (SNPs) were identified using SAMtools (Version 1.2) and BCFtools (Version 1.2). SNPs were filtered according to the following criteria: minimum allele frequency (MAF) ≥ 0.05, genotype call rate ≥ 80%, and P-value of the Hardy–Weinberg equilibrium (HWE) test ≥ 0.01. In addition, loci with significant copy number variation signals were excluded. Genome-wide screening of multinucleotide polymorphism (MNP) marker loci was performed using an MNP marker screening software developed for plants by the research team led by Professor Peng Hai from Jianghan University (Software Copyright Registration Number: 2019SR0838875).

The screening protocol was as follows: All the genome segments containing SNPs were scanned using a 300-bp sliding window with a 1-bp step size, and over 600 windows were screened. According to the criteria established in Liu et al. (2024a), regions with more than three SNP loci and a discriminative power (DP) ≥ 0.2 were designated as candidate MNP marker loci for *P. cyrtonema*. For each candidate marker, the amplicon length was restricted to 200–300 bp to prevent competitive amplification among different markers in the same reaction. Meanwhile, candidate MNP markers were required to be evenly distributed across the genome. The optimal species-specific loci were retained as the definitive MNP marker loci for *P. cyrtonema*.

### 2.4 Library construction via multiplex PCR amplification

Primers were designed and synthesized based on the screened *P. cyrtonema* MNP marker identification loci. These primers were then mixed in equal volumes to prepare a Primer Panel Mix. Subsequently, an HTS library was constructed via two rounds of PCR amplification.

#### 2.4.1 First-round PCR Amplification

The total volume of the first-round PCR reaction system was 30 µL, consisting of 4 µL of Primer Panel Mix, X µL of DNA template (20–200 ng), 10 µL of GenoPlexs 3 × T Master Mix, and (16−X) µL of double-distilled water (ddH_2_O). The PCR cycling program was as follows: pre-denaturation at 95 °C for 3 min, followed by 15 cycles of denaturation at 95 °C for 20 s, and combined annealing and extension at 60 °C for 4 min. After the cycling steps, a final extension was performed at 72 °C for 4 min, and the reaction was held at 4 °C. The first-round PCR products were collected and purified using a bead-based method.

#### 2.4.2 Second-round PCR amplification

The total volume of the second-round PCR reaction system was also 30 µL, containing the bead-purified product from the first round of PCR, 10 µL of GenoPlex 3 × T Master Mix, 16 µL of ultrapure water, 2 µL of P5 barcode, and 2 µL of P7 barcode. The PCR was conducted under the following conditions: denaturation at 95 °C for 3 min, followed by eight cycles of denaturation at 95 °C for 15 s, annealing at 58 °C for 15 s, and extension at 70 °C for 30 s. A final extension step was carried out at 72 °C for 5 min, and the reaction was then cooled to 4 °C.

After the library passed quality verification via gel electrophoresis and Qubit fluorometry, it was sent to Novogene Bioinformatics Technology Co., Ltd. (Beijing, China) for sequencing. The genotypes of the MNP loci were analyzed and quantified using the MLMNP cultivar analysis software (Software Copyright Registration Number: 2025SR0041458), and the combination of genotypes across all loci represented the genotyping result of the MNP markers. Based on the multiplex PCR amplification, HTS, and bioinformatics analysis, the MNP marker method for *P. cyrtonema* was initially established.

### 2.5 Evaluation of the MNP marker method

To further evaluate the reproducibility and accuracy of the selected MNP markers, a reproducibility test was conducted on the 30 tested accessions. This test comprised two independent experiments, which were performed by different operators and used different batches of reagents and different instruments. Comparative analysis was performed on the two sets of experimental data obtained for each sample to be tested. The genotyping accuracy rate was calculated using the formula: accuracy rate = 1 − (1 − reproducibility)/2.

### 2.6 Application of the MNP marker method for *P. cyrtonema* cultivar identification

Based on the sequencing library construction and genotyping protocols established in Section 2.4, the screened MNP markers were applied to the identification of 80 collected *P. cyrtonema* accessions. Pairwise comparisons of the core MNP sequences were conducted across all accessions. Accession pairs with identical sequences at the same MNP loci were classified as the same genotype. Genetic similarity (GS) was calculated as the number of identical MNP sequences between two accessions divided by the total number of core MNP sequences (Liu et al., 2023b). Visualization of GS data was performed using the Metware Cloud Platform (Wuhan Maiteville Biotechnology Co., Ltd., Wuhan, China; https://cloud.metware.cn), accessed on June 3 2025. Based on the core MNP sequences and pairwise GS values, cluster analysis and PCA were performed using the Metware Cloud Platform (accessed on June 4 2025). The performance of the MNP markers for the 80 P*. cyrtonema* accessions was evaluated in terms of detection efficiency, detection rate, accuracy, discriminatory power, and polymorphism.

## 3 Results

### 3.1 Genome sequencing

A *de novo* assembly of the reference genome data was performed, and contig sequences longer than 250 bp were retained for subsequent analyses. In total, 32 GB of reference genome sequencing data was generated, which was assembled into 3,444,851 contigs (each > 250 bp). Among these contigs, the maximum length was 20,786 bp, with an overall GC content of 40.79%.

RRGS was conducted on 30 P*. cyrtonema* accessions collected from different geographical regions, yielding approximately 93.7 GB of clean data. To assess the quality of the sequencing data, we mapped the sequencing reads to the assembled reference genome. The average number of total reads per accession was 26,023,536, with an average mapping rate of 57.5% for the sequencing reads (**Supplementary Table S2**). Given the sufficient data volume and normal GC content distribution, after excluding samples with low mapping rates, the sequencing data were used for further MNP marker screening.

### 3.2 Screening of MNP marker sites and genetic information of *P. cyrtonema*

Based on the RRGS data of the 30 P*. cyrtonema* accessions, most of these accessions harbored over 10,000 MNP marker loci (**Supplementary Table S2**). Following the MNP marker screening protocol described in Section 2.3, the MNP marker loci detected from RRGS were evaluated for their discriminatory power. Through this evaluation, 1,042 MNP markers were identified as candidate loci for *P. cyrtonema* cultivar identification. After the design and evaluation of multiplex PCR amplification primers, 505 MNP marker loci were retained (**Supplementary Table S3**), encompassing 4,741 mutation sites.

**Figure 2:**
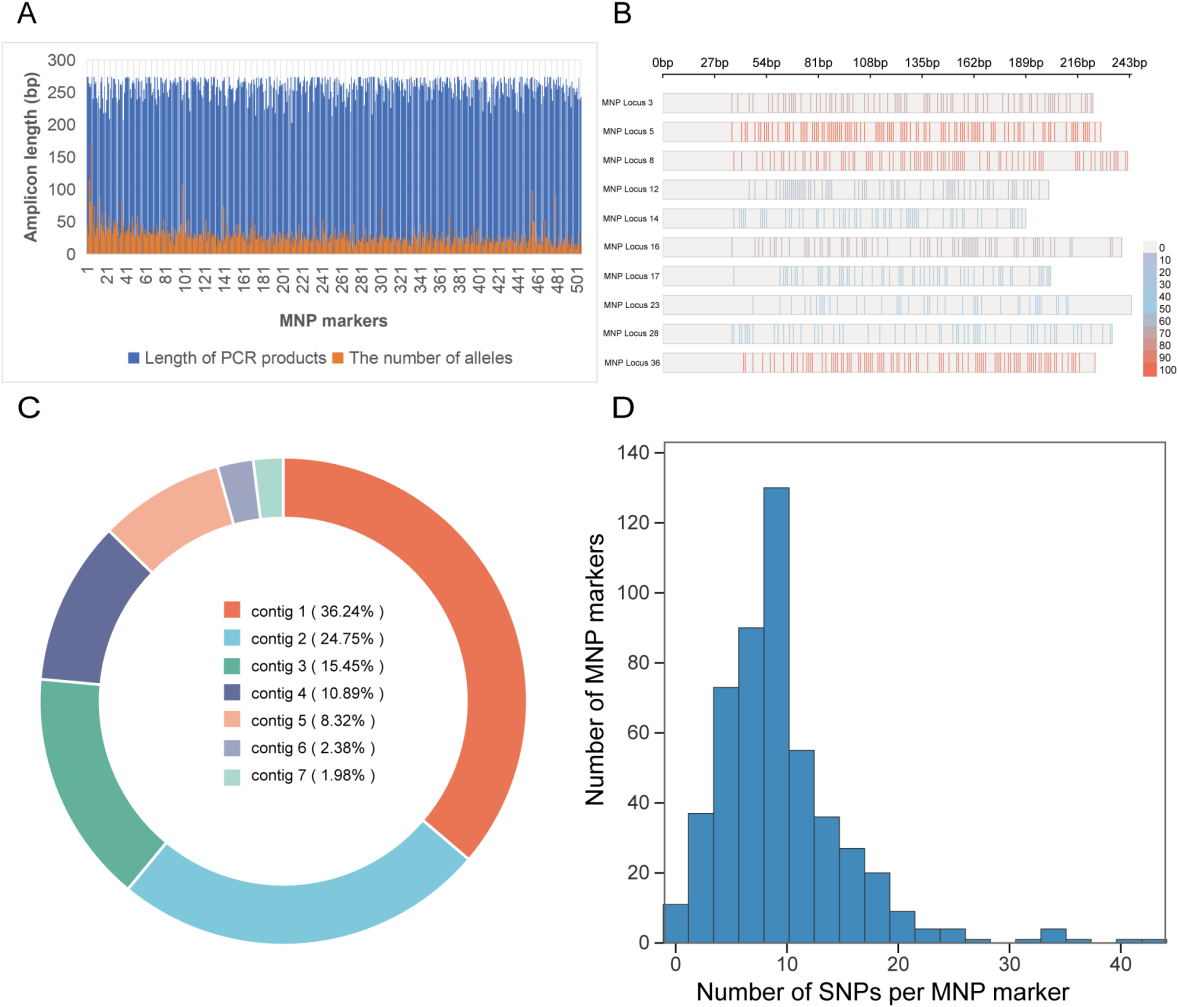
Characteristics of 505 multiple nucleotide polymorphism (MNP) markers. **(a)** Statistical analysis of sequence lengths and the number of alleles per MNP locus for the 505 MNP markers; **(b)** The number of SNPs and mutation frequencies within the 10 MNP loci. The closer the color is to red, the higher the nucleotide diversity. **(c)** Statistical analysis of the distribution of the 505 MNP markers across chromosomes contigs; **(d)** Number of single-nucleotide polymorphism (SNP) loci within each MNP locus.

The amplicon lengths of the screened 505 MNP markers ranged from 203 to 274 bp (**Figure 2a**; **Supplementary Table S3**). The number of alleles detected per MNP marker ranged from 7 to 171, with an average of 28.95 allelic genotypes per marker. Of them, 496 MNP markers contained more than 10 allelic genotypes (**Figure 2a**; **Supplementary Table S3**). **Figure 2b** shows the number of SNPs and mutation frequencies within 10 randomly selected MNP loci, demonstrating abundant nucleotide polymorphism characteristics. The markers were primarily distributed across seven chromosomes contigs (**Figure 2c**). Each MNP marker harbored 1–44 SNP loci, with an average of 9.4 SNPs per marker (**Figure 2d**). In addition, **Supplementary Table S4** covers 505 representative MNP loci, highlighting the types of nucleotide variations (A/T/C/G) among different germplasms and providing corresponding annotations for locus IDs and polymorphism types.

### 3.3 Establishment and evaluation of the MNP marker assay workflow

**Figure 3:**
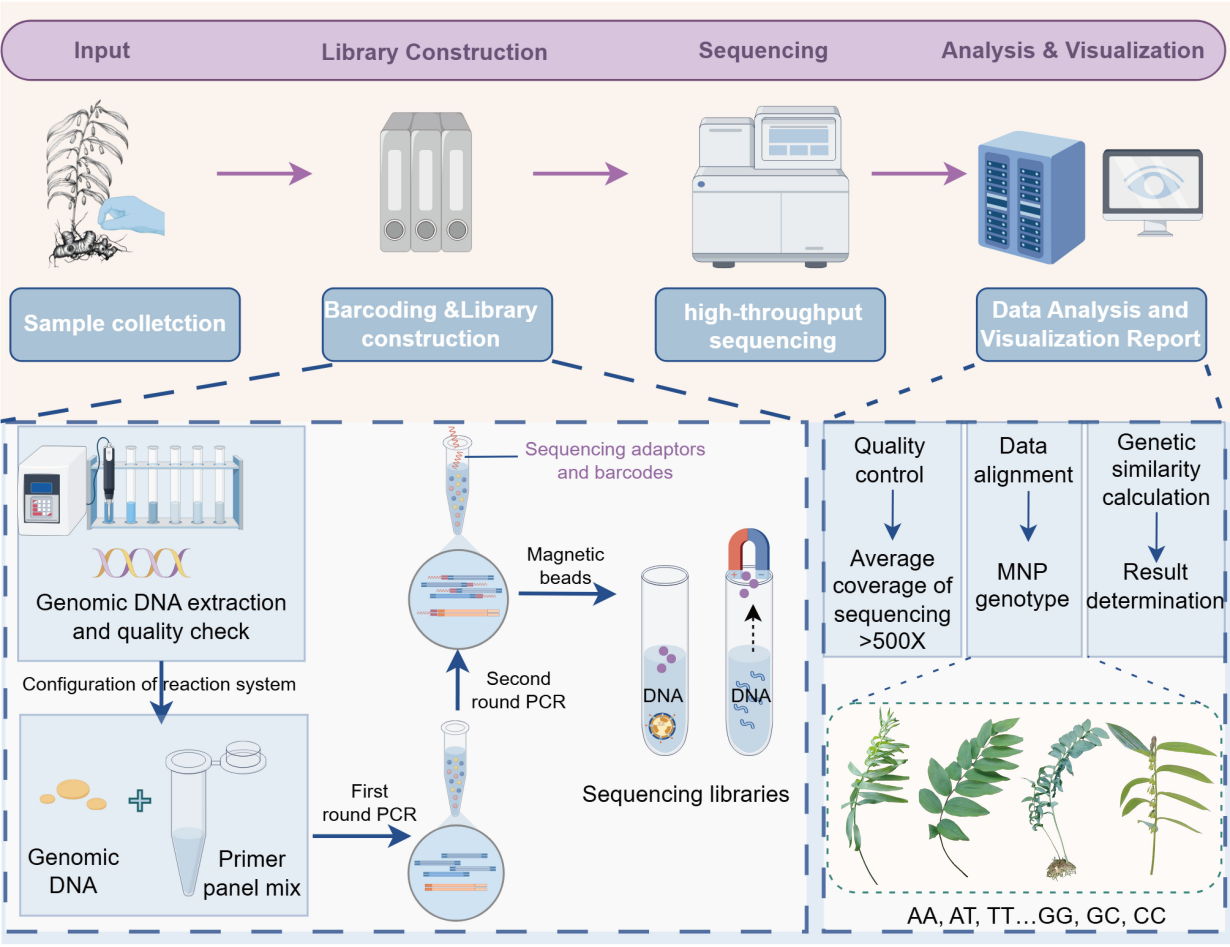
Genotyping workflow for *Polygonatum cyrtonema* accessions using MNP markers. This figure was drawn with Figdraw.

In accordance with the protocol specified in the Chinese National Standard Plant Cultivar Identification—MNP Markers (GB/T 38551-2020), an experimental workflow for detecting P*. cyrtonema* MNP marker loci was established. First, HTS libraries were constructed via two rounds of PCR amplification, using genomic DNA from 30 representative *P. cyrtonema* accessions as templates, with qualified quality inspection. The first round of PCR was used to enrich target fragments. Meanwhile, the second-round introduced adapters compatible with Illumina sequencing and specific DNA barcodes. After obtaining the sequencing libraries, they were sequenced on an HTS platform. Genotyping of MNP markers was performed using analytical software (**Figure 3**).

For the 30 accessions, 14,460 marker loci were detected, with an average sequencing coverage depth of 2654.90× per accession. The number of MNP markers detected per accession ranged from 308 to 505, out of 505 target markers, with an average of 482 markers detected per sample. Analysis of the detection rate [calculated as (number of detected loci/total target loci)] for the 505 MNP loci across the 30 accessions showed that the detection rates of DH-52, DH-53, and DH-54 were below 70%. Meanwhile, those of the remaining 27 accessions exceeded 96% (**Table 1**). Overall, the MNP markers exhibited high detection efficiency.

**Table 1:** Basic sequencing information of multiple nucleotide polymorphism marker libraries for 30 *Polygonatum cyrtonema* accessions.

Sample ID	Total sequencing reads	Number of detected loci	Detection rate	Average locus cverage
DH-01	1566831	503	99.60%	1959.91
DH-02	2479196	504	99.80%	2968.27
DH-03	2645174	503	99.60%	3217.45
DH-09	2274221	494	97.82%	2857.68
DH-21	1895372	504	99.80%	2574.92
DH-25	2113145	502	99.41%	2545.39
DH-31	2076447	499	98.81%	2989.8
DH-36	1419326	498	98.61%	2019.62
DH-37	1574484	501	99.21%	2128.45
DH-38	1926518	502	99.41%	2251.9
DH-39	1906651	492	97.43%	894.74
DH-40	2354870	501	99.21%	2943.19
DH-41	1941975	505	100.00%	2838.59
DH-42	1468994	503	99.60%	1325.06
DH-43	2083981	502	99.41%	2751.75
DH-44	1357477	496	98.22%	1923.23
DH-45	1802192	503	99.60%	2381.17
DH-46	2249200	500	99.01%	3386.63
DH-47	2285874	497	98.42%	2982.92
DH-48	2388324	504	99.80%	3243.45
DH-49	2751170	500	99.01%	3686.78
DH-50	2140281	489	96.83%	3231.03
DH-51	1888631	502	99.41%	2469.06
DH-52	1671170	308	60.99%	3608.28
DH-53	1240654	316	62.57%	2086.57
DH-54	2571645	327	64.75%	4413.4
DH-55	2881190	503	99.60%	3117.24
DH-56	1192105	500	99.01%	596.5
DH-57	2377136	505	100.00%	2929.57
DH-58	2732745	497	98.42%	3324.59

To evaluate reproducibility, two batches of libraries were constructed from the same DNA sample of each of the 30 accessions. The number of differential markers between the two batches was counted. Among the 14,425 marker loci compared in this study, 14,392 were reproducible. This resulted in a genotyping reproducibility of 99.78% and a genotyping accuracy of 99.89% (**Supplementary Table S5**). MNP markers can be effectively used for the molecular identification of *P. cyrtonema* and the exploitation of its germplasm resources.

### 3.4 Application of MNP markers

**Figure 4:**
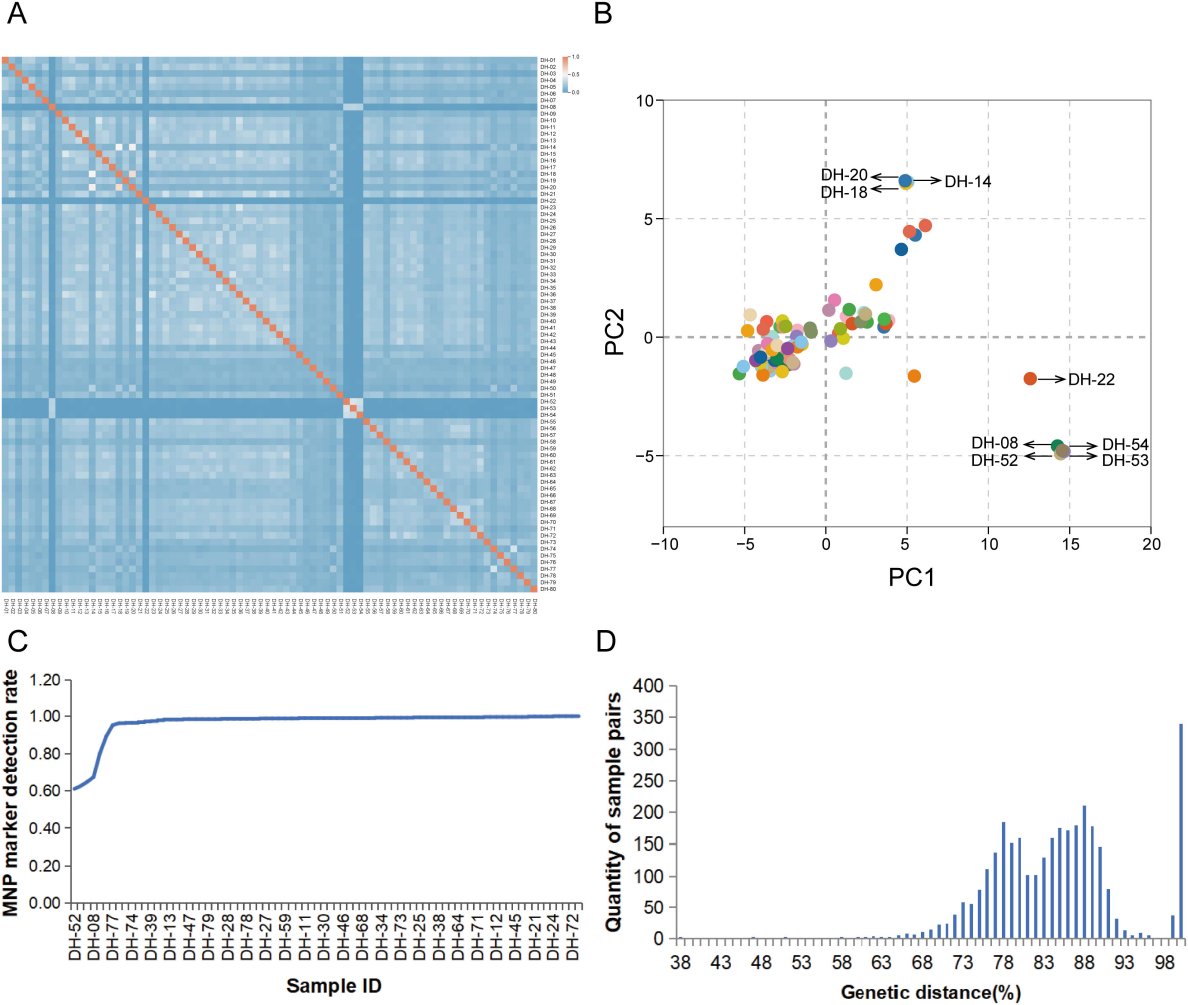
Application results of MNP markers for 80 *Polygonatum cyrtonema* accessions. **(a)** Genetic similarity heatmap of pairwise comparisons among the 80 P*. cyrtonema* accessions. The closer the color is to orange, the higher the similarity; **(b)** PCA of *P. cyrtonema* accessions based on genetic similarity. **(c)** Distribution of detection rates for the 505 MNP markers in *P. cyrtonema*. **(d)** Distribution of MNP marker differentiation ratios among the 80 P*. cyrtonema* accessions.

To validate the established MNP method, GS analysis was conducted on 80 P*. cyrtonema* accessions. The heatmap (**Figure 4a**) showed that the GS values between any two accessions among the 80 ranged from 0 to 62.45% (**Supplementary Table S6**). Among these pairs, DH-18 and DH-20 exhibited the highest GS (62.45%), with 181 differential loci identified between them. Based on the genotyping results of the 505 MNP markers, PCA and unweighted pair group method with arithmetic mean cluster analysis were performed on the 80 accessions. The PCA results (**Figure 4b**) indicate that a subset of accessions, including DH-14, DH-18, DH-20, DH-08, DH-52, DH-53, DH-54, and DH-22, could be significantly distinguished from the remaining accessions, exhibiting substantial genetic divergence.

The MNP markers and their associated technical system were evaluated based on MNP genotyping data from the 80 P*. cyrtonema* accessions. During this experiment, 80 × 505 = 40,404 marker loci were detected and analyzed in a single run, demonstrating the high level of efficiency of the technology. Analysis of the MNP marker sequencing data for the 80 accessions showed that the average coverage depth of the markers reached 2800×, with an average of 488.7 markers detected per accession and a mean marker detection rate of 96.77%. The distribution of overall detection rates is shown in **Figure 4c**. The results from the reproducibility test of the 80 accessions (**Supplementary Table S7**) indicated that 38,947 MNP markers were compared, with a reproducible locus ratio of 99.80% and a genotyping accuracy of 99.90%. These results confirm the high level of accuracy of the developed MNP markers for *P. cyrtonema*. Pairwise comparisons were conducted among the 80 P*. cyrtonema* accessions to count the number of differential MNP markers. This yielded 3,160 pairwise comparison results. Each pair of test accessions had an average of 394.85 differential marker loci, with an average differentiation ratio of 84.03%. The distribution of differentiation ratios is shown in **Figure 4d**. Polymorphism information of the MNP markers was further calculated based on these accessions. The average number of allelic genotypes for the MNP markers was 28.95 ± 15.11, and the polymorphism information content (PIC) value was 0.73 ± 0.16 (**Supplementary Table S8**). These results demonstrate that the screened MNP markers for *P. cyrtonema* exhibit high polymorphism and can significantly distinguish *P. cyrtonema* cultivars.

**Figure 5:**
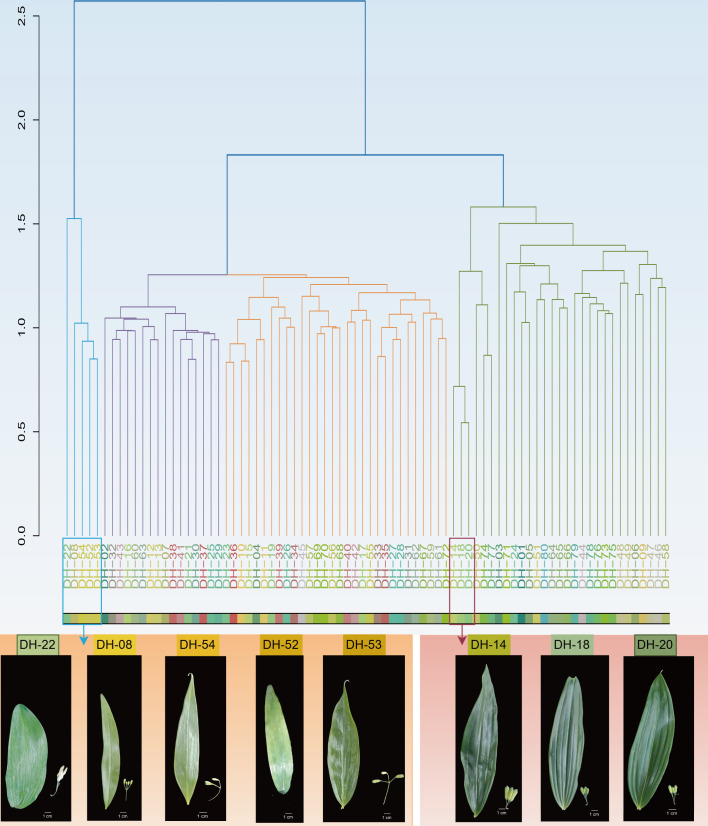
Phylogenetic tree of 80 *Polygonatum cyrtonema* accessions constructed based on the sequences of 505 polymorphic nucleotide loci.

Cluster analysis showed that the 80 P*. cyrtonema* accessions were primarily grouped into four major clades (**Figure 5**). The similarity within the same clade ranged from 10.24 to 62.45%. Accessions falling within this range could be classified as different cultivars under the same lineage. In contrast, accessions with a GS value of less than 10.24% could be distinguished as distinct lineages. Accessions within the same sub-clade with high mutual similarity shared similar phenotypic traits. DH-14, DH-18, and DH-20, all collected from Anhua, Hunan Province, had oblong leaves with a subcoriaceous texture, prominent leaf veins, and short pedicels. DH-08, DH-52, DH-53, and DH-54, primarily collected from the Sichuan Province, had lanceolate leaves with a submembranous texture and long pedicels. A unique *P. cyrtonema* accession (DH-22) was identified, characterized by broadly ovate-elliptic leaves. Its maximum GS with any other accession was 2.03%. These clustering results were generally consistent with the outcomes of the PCA.

## 4 Discussion

*P. cyrtonema* is rich in agavin-type fructans, which have the dual functions of energy supply and chronic disease prevention (Si et al., 2025), which implies that it is extensively used in clinical practice. The Chinese Pharmacopoeia (2025 Edition) records more than 30 Chinese patent medicines containing *P. cyrtonema*. Due to its abundant nutrients, *P. cyrtonema* is widely used in the development of functional foods and dietary supplements (Cheng et al., 2021). It also has favorable antioxidant and anti-aging properties, which have been applied in the development of skincare products (Bai et al., 2021; Wang et al., 2023). Therefore, *P. cyrtonema* has become one of the most consumed plants with dual use as food and medicine among the Chinese population. However, *P. cyrtonema* has a wide distribution and has considerable variation in morphological characteristics. The content of polysaccharides, that is, its main bioactive component, can differ by more than 15% among different regions or cultivars. Therefore, issues related to cultivar identification and superior germplasm have become major bottlenecks restricting the current development of the *P. cyrtonema* industry.

In this study, a highly efficient identification system for *Polygonatum cyrtonema* was successfully developed based on HTS, encompassing 505 core MNP markers. The MNP markers had an average of 28.95 ± 15.11 allelic genotypes per locus, with a genotyping accuracy as high as 99.9%. This marker panel has strong discriminatory power, which forms the basis for generating a unique molecular ID for each cultivar. The PIC value of the MNP markers was 0.73 ± 0.16. A PIC value > 0.5 is generally indicates that a marker is highly polymorphic and -informative (Brhane and Hammenhag, 2024). These evaluation metrics are substantially higher than those of marker types reported in previous studies. In prior research, 96 P*. cyrtonema* accessions were analyzed using 10 SSR markers, yielding an average of 6.0 alleles per locus and a mean PIC value of 0.6430 (Liu et al., 2023a). ISSR markers used to analyze 118 P*. cyrtonema* individuals resulted in an average number of alleles (Na) of 5.916 and a mean PIC value of 0.585 (Liu et al., 2020). Another study investigating the genetic diversity of 50 *Polygonatum* accessions using EST-SSR and SRAP markers reported an allele number (Na) ranging from 1 to 2, with PIC values spanning 0.508–0.945 (Feng et al., 2020). These findings confirm that the MNP marker system exhibits high efficiency in identifying subtle genetic differences among *P. cyrtonema* cultivars/germplasm.

*P. cyrtonema* is a perennial herbaceous plant that primarily reproduces asexually via buds. Under natural conditions, sexual reproduction via seeds is highly inefficient, leading to substantial genetic differences among cultivars. Clustering results indicated a certain correlation between different *P. cyrtonema* lineages and their geographical distribution. However, introduction and cultivation across regions have caused genetic admixture, generating additional variations. Unlike *Agaricus bisporus*, where GS values among cultivars all exceed 66.7% (Liu et al., 2025a), our results showed that the highest GS value between any two *P. cyrtonema* cultivars was only 62.45%. This indicates that *P. cyrtonema* has extremely rich genetic diversity, which has also been confirmed in several previous studies (Feng et al., 2020; Ji et al., 2020; Hai et al., 2025). The phylogenetic relationships and intrageneric taxonomy within the genus *Polygonatum* have long been controversial (Wang et al., 2022; Xia et al., 2022). Based on morphological and molecular phylogenetic studies, some earlier classification systems placed *Polygonatum* in Convallariaceae, Ruscaceae, or Liliaceae. However, it is now classified under Asparagaceae (Rudall et al., 2000; Kim et al., 2012; Wang et al., 2022). Regarding intraspecific relationships within *Polygonatum*, the genus has been divided into two sections, three sections, or eight series (Baker, 1875; Tamura, 1993). Based on evidence from morphology, chromosome karyotypes, and chloroplast genomes, the most widely accepted classification system currently divides *Polygonatum* into three sections: sect. *Polygonatum*, the monotypic sect. *Sibirica*, and sect. *Verticillata* (Floden and Schilling, 2018; Zhao et al., 2019a). However, genetic analyses based on chloroplast genomes have limited information content and cannot reflect most traits or the genetic diversity of organisms. Although these studies have clarified the phylogenetic relationships of some *Polygonatum* species, the phylogenetic relationships among genera within Tribe Polygonateae and among certain species of *Polygonatum* remain unclear. MNP markers based on WGS are expected to resolve this long-standing challenge for researchers. Currently, we are conducting germplasm resource collection of *Polygonatum* species such as *P. sibiricum* and *P. odoratum*. Subsequently, we will proceed with the development of MNP markers for the genus *Polygonatum*.

In the circulation and application of Chinese medicinal materials, a single name of a medicinal material often corresponds to multiple botanical origins. Coupled with the similarity in plant morphology, this exacerbates the issue of adulteration and misidentification among different medicinal plants. Therefore, establishing an efficient and practical cultivar identification method is crucial for resolving the confusion surrounding Chinese medicinal material varieties. Compared with traditional morphological markers, MNP markers are not affected by factors such as environment and growth stage, offering a shorter identification cycle and higher accuracy. This addresses the drawbacks of morphological identification, such as strong subjectivity and long cycles. In contrast to SSR markers, MNP technology has advantages in high throughput and automation. SSR analysis typically requires gel electrophoresis, involving cumbersome operations, low throughput, and difficulty in standardization across laboratories (Amiteye, 2021). In contrast, MNP markers, based on HTS reads, enable automation, digitization, and standardization, with precise and reproducible results. This makes them particularly suitable for large-scale germplasm resource surveys and commercial applications. Compared with SNP chips, MNP markers offer greater cost advantages and flexibility (Liu et al., 2024a). The development of an SNP chip incurs high fixed costs, and once designed, the loci cannot be updated. In contrast, the MNP method, which is sequencing-based, eliminates the need for chip hardware costs. The MNP panel (505 loci) can be flexibly expanded or reduced in locus number according to future needs, with relatively low costs for iterative updates. Furthermore, we conducted a comprehensive comparison between the MNP platform and three widely used genotyping methods (SSR, genotyping by sequencing (GBS), and SNP chips; **Supplementary Table S9**). The MNP marker technology integrates the high density of SNPs, high informativeness of SSRs, and stability of codominant markers while avoiding the limitations of the insufficient informativeness of single SNPs, high development costs of SSRs, and narrow sampling scope of GBS.

This study preliminarily validated the effectiveness of MNP markers for the identification of *P. cyrtonema* cultivars. However, the tested sample size was relatively limited, failing to cover all known landraces, wild populations, and elite breeding lines of *P. cyrtonema*. Although the currently developed 505 core MNP loci can effectively distinguish existing samples, their distribution uniformity and density across the genome need to be further improved. For example, the MNP marker call rates of DH-52, DH-53 and DH-54 were relatively low, which may be attributed to the poor compatibility of the MNP panel designed based on the core population. Increasing marker density, especially in gene-enriched regions or regions associated with important traits, is expected to further enhance identification accuracy and tap the potential of discovering functional markers. Similar to most previous reports regarding the determination of essentially derived varieties, in this study, we did not establish a specific GS threshold for *P. cyrtonema* cultivar discrimination because of the high genetic variability of the species. Based on the achievements and limitations of this research, we developed the MNP-based identification system into a commercial kit (**Supplementary Figure 2**). This is intended for application in larger sample sizes and a broader range of genetic diversity.

Notably, the chromosome-level genome of *P. cyrtonema* has not been released, nor has the ploidy of its germplasms been clearly characterized, which has precluded the analysis of population genetic differentiation. However, MNP markers have been demonstrated to enable the distinct differentiation of diploid, triploid and tetraploid accessions in grapes (Liu et al., 2024a), providing a valuable reference for our research. With the release of more genomic data in the future, the population genetic structure of *P. cyrtonema* is expected to be elucidated with greater clarity. In the future, we will systematically collect *P. cyrtonema* germplasm resources from different ecological regions and various sources, including wild, cultivated, landraces, and improved varieties nationwide. In addition, the applicability of this method to the closely related species of *P. cyrtonema* and its processed products will be further verified. Using this MNP system, we will conduct large-scale genetic evaluations to establish a more comprehensive and authoritative cultivar identification database and reference map. Ultimately, we aim to determine the optimal GS threshold, enabling the kit to address issues related to new plant variety protection, such as cultivar rights infringement and disputes. Based on establishing a larger-scale population and acquiring accurate phenotypic data, we will use this MNP marker system to perform genome-wide association studies. This will facilitate the mining of loci significantly associated with important agronomic/medicinal traits, such as medicinal component content, yield, and stress resistance (Tibbs Cortes et al., 2021), promoting the molecular design breeding of *P. cyrtonema*. The technical route and bioinformatics pipeline used in this study are equally applicable for developing MNP marker systems for other important medicinal plants facing similar variety confusion issues, such as other *Polygonatum* species, *Panax ginseng*, *Panax notoginseng*, and *Dendrobium* spp. Therefore, MNP technology is expected to become a universal and efficient platform in the fields of medicinal plant germplasm resource identification and molecular breeding.

## 5 Conclusions

In this study, a high-performance DNA fingerprinting authentication system based on MNP markers was developed and validated for *P. cyrtonema*, which comprises 505 evenly distributed species-specific loci. This system achieved a genotypic line-level authentication accuracy of 98.7% and simultaneously features the advantages of low detection cost and high throughput. Validated with diverse geographical populations and replicate samples, the system provides a reliable tool for the authentication of Chinese medicinal materials, cultivar registration, and germplasm resource management. Compared with existing authentication methods (SSR, SNP chips, and ITS sequencing), this MNP marker platform can achieve a favorable balance among detection efficiency, cost, and applicability, making it suitable for large-scale industrial application. Future research will focus on expanding the marker panel to cover international germplasm resources and integrating it with metabolomic markers for the authentication of *P. cyrtonema* cultivars with different ploidy levels and its processed products.

## Data Availability

The data presented in the study are deposited in the Genome Sequence Archive (Genomics, Proteomics & Bioinformatics 2025) in National Genomics Data Center (Nucleic Acids Res 2025), China National Center for Bioinformation / Beijing Institute of Genomics repository, accession number CRA038989. Please access it from the following link: https://ngdc.cncb.ac.cn/gsa/browse/CRA038989.
