# Supplementary material for: Construction and application of multiple nucleotide polymorphism-based DNA fingerprinting for Polygonatum cyrtonema identification
Source: Front Plant Sci. 2026 Feb 27;17:1758042. doi: 10.3389/fpls.2026.1758042 (PMC12982458; doi:10.3389/fpls.2026.1758042)
Supplement: Supplementary file 1 [file DataSheet1.pdf]

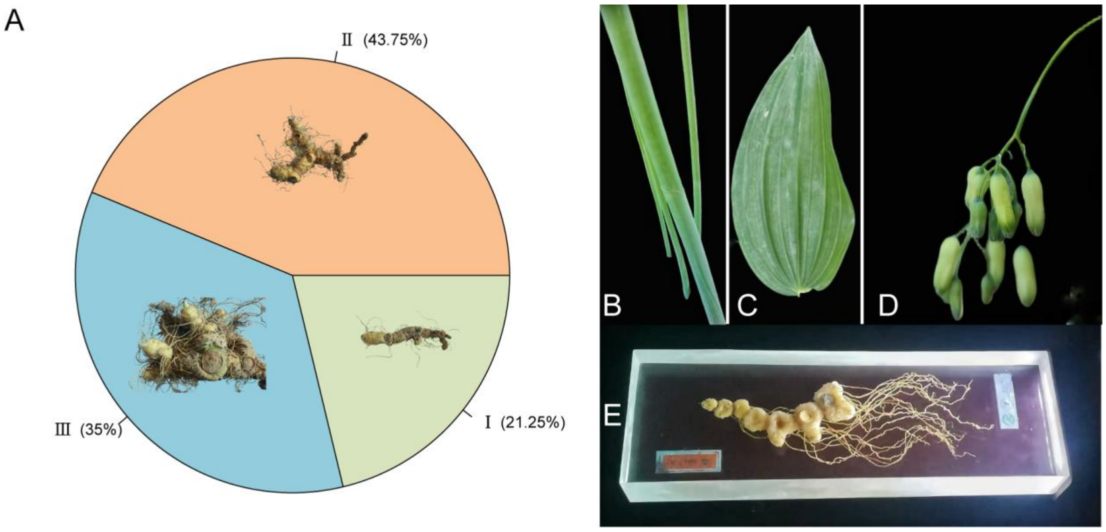

Figure S1:Key morphological features of *P. cyrtonema* (A) Root system density statistics, (B)Stem, (C)Leaf, (D)Flower, (E)Rhizome

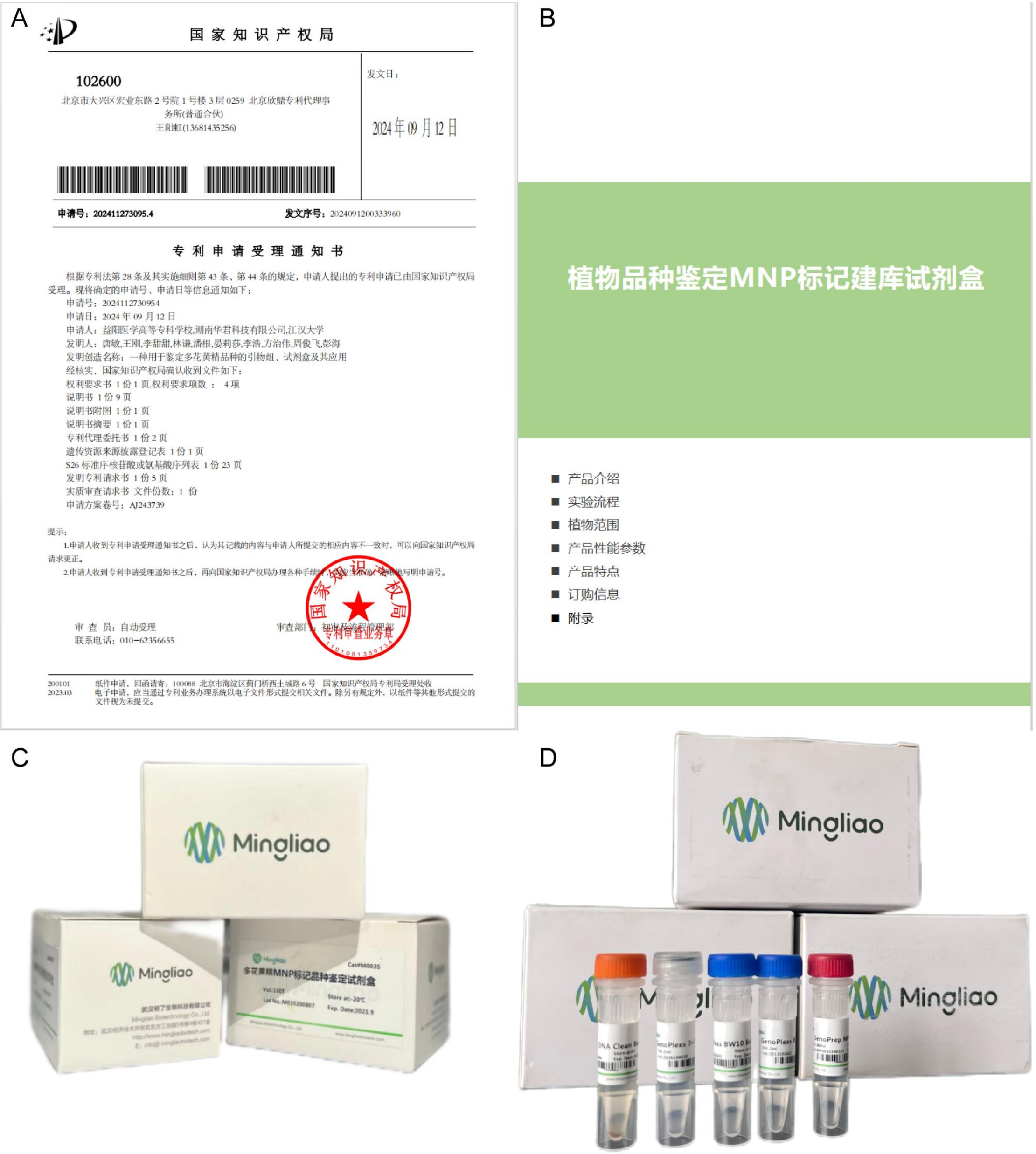

Figure S2:Cultivar identification kit for *Polygonatum cyrtonema* based on multinucleotide polymorphism (MNP) markers (A) Patent of the cultivar identification kit for polygonatum cyrtonema based on MNP markers (B) Instruction manual of the kit (C) Outer packaging of the kit (D) Reagent composition of the kit
